# Immunomodulatory Activity of Recombinant Ricin Toxin Binding Subunit B (RTB)

**DOI:** 10.3390/ijms140612401

**Published:** 2013-06-13

**Authors:** Wensen Liu, Na Xu, Hongyan Yuan, Songyan Li, Linna Liu, Zhaoyang Pu, Jiayu Wan, Huiwen Wang, Yaping Chang, Ruisheng Li

**Affiliations:** 1 Institute of Military Veterinary, Academy of Military Medical Sciences, Zoonosis Prevention and Control Key Laboratory, Changchun 130122, China; E-Mails: xuna206@aliyun.com (N.X.); lisongyan888@163.com(S.L.); liulinna@126.com (L.L.); puzhaoyang@126.com (Z.P.); jywan_1973@163.com (J.W.); 2 Department of Immunology, Norman Bethune College of Medical Science, Jilin University, Changchun 130021, China; E-Mails: yuanhongyan@126.com (H.Y.); wanghuiwen@126.com (H.W.); 3 Dean’s Office, Jilin Medical College, Jilin 132013, China; 4 Animal Laboratory Center, 302 Hospital of People’s Liberation Army, Beijing 100039, China

**Keywords:** RTB, macrophage, lymphocyte proliferation, Th1 cell, cytokine

## Abstract

Ricin toxin binding subunit B (RTB) is one of the subunits of the ricin protein. RTB has been used as adjuvant, but little is known about its mechanism. In this study, we found that RTB increased not only nitric oxide (NO) release, but also tumor necrosis factor (TNF)-α and interleukin (IL)-6 production in mouse macrophage cell line RAW264.7 cells. They subsequently exhibited enhanced ConA-induced T-cell and LPS-induced B-cell proliferative responses. We also examined the cytokines that were produced from splenocytes following *in vitro* RTB administration. Increased levels of IL-2, interferon (IFN)-γ and TNF-α were observed, while IL-4 and IL-5 were unaffected. These results demonstrate that recombinant RTB can act on the immune system and activate T-cells by introducing a Th1 immune response. Th1 cells might be the primary cellular target affected by RTB. Our results suggest that the recombinant RTB can promote the activation of macrophages and has a beneficial effect on immunomodulatory activity.

## 1. Introduction

Ricin is a heterodimeric protein toxin produced by the seeds of *Ricinus communis*. Ricin toxin belongs to the type II ribosome-inactivating protein family (type II RIPs). It is composed of A and B chains that are joined by a disulfide bond [1]. The B-chain (RTB) is a lectin that mediates toxin internalization; specifically, RTB’s terminal galactose residues bind to glycolipids and glycoproteins that are on the surface of almost every cell type [2,3]. The A-chain of the ricin toxin (RTA) is not toxic without the B-chain (RTB), as RTB is required for the delivery of RTA into the cytosol of target cells [4]. RTB alone is also not toxic, as it exhibits no activity other than binding to galactose/*N*-acetylgalactosamine. Several reports have demonstrated that RTB can function as a carrier molecule and that a properly folded RTB is expressed in transgenic plants [5,6]. Furthermore, RTB has been used as a target for antibody-based intervention therapeutics against infectious disease, and the enhancement of vaccines has also been demonstrated [7–9]. However, aside from these studies, the mechanism and immunogenicity of RTB remains poorly understood.

RTB was used as adjuvant primarily by their ability to stimulate humoral immune responses. However, studies of the effects of RTB on cellular immunity have been neglected. Cell-mediated immune responses involve the activation of macrophages and the release of cytokines in response to the antigen. When macrophages become activated, they release inflammatory mediators, such as nitric oxide (NO) and tumor necrosis factor (TNF-α) and interleukin (IL-6) to inhibit tumor formation and microbial infections [10].

In the present study, we investigate the functional activation of RTB by analyzing the production of NO, TNF-α and IL-6 from mouse macrophage cell line RAW264.7 cells. We also analyzed mitogen response induced by RTB in splenocytes and splenocyte cytokine expression.

## 2. Results

### 2.1. The Purification of Recombinant RTB

**Figure 1:**
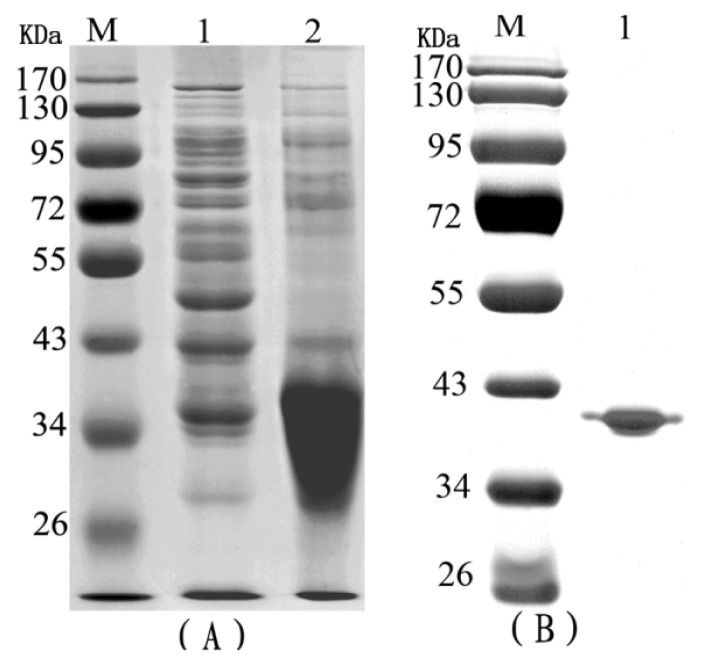
Expression and purification of recombinant ricin toxin B subunit (RTB). (**A**) Lane M contains molecular weight markers (Invitrogen); lane 1, bacterial strain BL21(DE3) pLys extract; lane 2, cell extract containing expressed RTB protein; (**B**) Lane M contains molecular weight markers (Invitrogen); lane 1, RTB protein after purification by Ni2^+^ affinity column chromatography.

Purified, denatured RTB proteins that were generated in *E. coli* were separated by SDS-PAGE (Figure 1A). The purified recombinant RTB was observed as a single band on the gel, and its molecular weight was approximately 34 kDa (Figure 1B).

### 2.2. Cell Viability Assay

**Figure 2:**
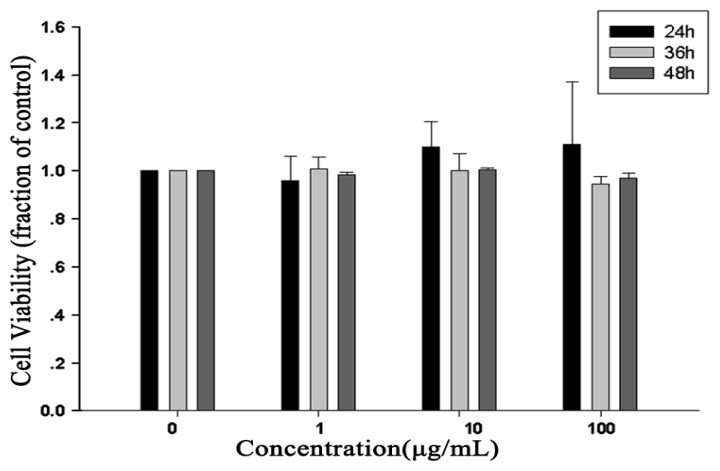
Effects of RTB on cell viability. RAW264.7 cells were treated with various concentrations of RTB for 24, 36 or 48 h, respectively. Cell viability was determined by alamarBlue assay, as described in Materials and Methods. Cell viability in absence of RTB treatment was taken as 100%. The results were expressed as the mean ± SD of three independent experiments.

Cell viability was evaluated to assess cytotoxicity. RAW264.7 cells were incubated with various concentrations of RTB. The results showed no cytotoxic effects on RAW264.7 cells compared to non-stimulated cells (Figure 2). Based on these results, the concentrations of RTB were used in the following experiments.

### 2.3. No Production

**Figure 3:**
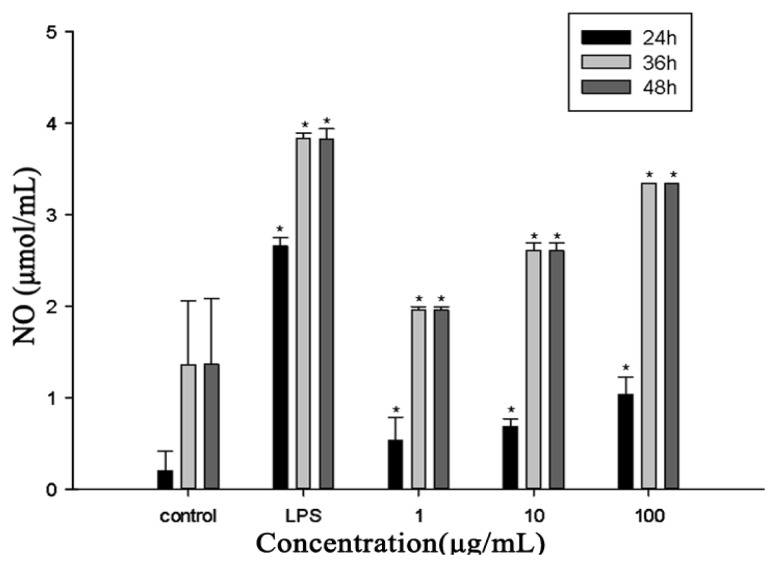
NO production in RAW264.7 cells treated with RTB. RAW264.7 cells were added with RTB (1, 10 and 100 μg/mL) or LPS (50 ng/mL) for 24, 36 and 48 h, respectively. NO production was measured by Griess reagent. Data are presented as the means ± SD of three replicates. *p < 0.01 compared to the control group.

The stimulation of RAW264.7 cells by RTB resulted in the NO production, which increased in a dose-dependent and time-dependent manner. The difference in NO production between RTB (1, 10 and 100 μg/mL) or LPS (50 ng/mL)-stimulated groups and the control group were statistically significant (Figure 3).

### 2.4. Cytokine Production of RAW264.7 Cells

**Figure 4:**
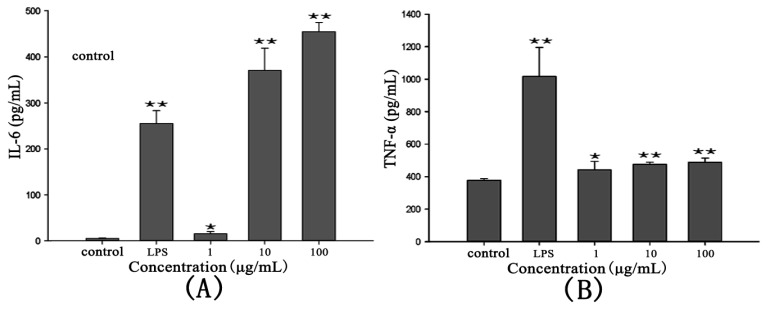
IL-6 and TNF-α production in RAW264.7 cells treated with RTB. (**A**) IL-6 production in RAW264.7 cells treated with RTB; (**B**) TNF-α production in RAW264.7 cells treated with RTB. RAW264.7 cells were added with RTB (1, 10 and 100 μg/mL) or LPS (50 ng/mL) for 24 h. TNF-α and IL-6 production was measured by ELISA. Data are presented as the means ± SD of three replicates. *p < 0.05, **p < 0.01 compared to the control group.

The production of TNF-α and IL-6 treated with RTB followed a tendency similar to that of NO release (Figure 4A,B). TNF and IL-6 release depended on the concentrations of RTB. The concentrations of TNF and IL-6 stimulated by RTB (1, 10 and 100 μg/mL) or LPS (50 ng/mL) were significantly higher than those in the control group.

### 2.5. The Proliferation of Mouse Splenocytes *in Vitro*

**Figure 5:**
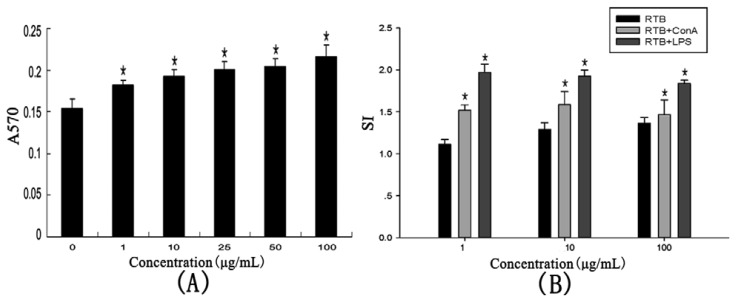
Effects of RTB on proliferation of mouse splenocytes *in vitro*. (**A**) Splenocytes were measured with treated RTB (from 0 to 100 μg/mL) for 48 h; (**B**) Splenocytes were measured with RTB (1, 10 and 100 μg/mL) in the presence of 2.5 μg/mL ConA or 5 μg/mL LPS for 48 h. The absorbance at 570 nm was measured. SI (RTB) = mean A570 of RTB/mean A570 of media control, SI (mitogen) = mean A570 of RTB + ConA or LPS/mean A570 of ConA or LPS. Values are the mean ± SD of three replicates. *p < 0.01 compared to the control group.

The effect on lymphocyte proliferation *in vitro* was investigated (Figure 5A). RTB was found to enhance splenocyte proliferation compared with control. The effect of RTB on the splenocyte proliferation was in a dose-dependent manner at concentrations from 1 to 100 μg/mL.

T-cell mitogen ConA and B-cell mitogen LPS were utilized to stimulate proliferation at concentration levels of 1, 10 and 100 μg/mL. In the presence of ConA or LPS, the treatment with the 1 to 100 μg/mL concentrations significantly enhanced the spleen index (Figure 5B). The response of splenocytes proliferation was not in a dose-dependent manner. This suggested that RTB could stimulate the proliferation of T-cells, as well as B-cells.

### 2.6. Spleen Cell Cytokine Production

**Figure 6:**
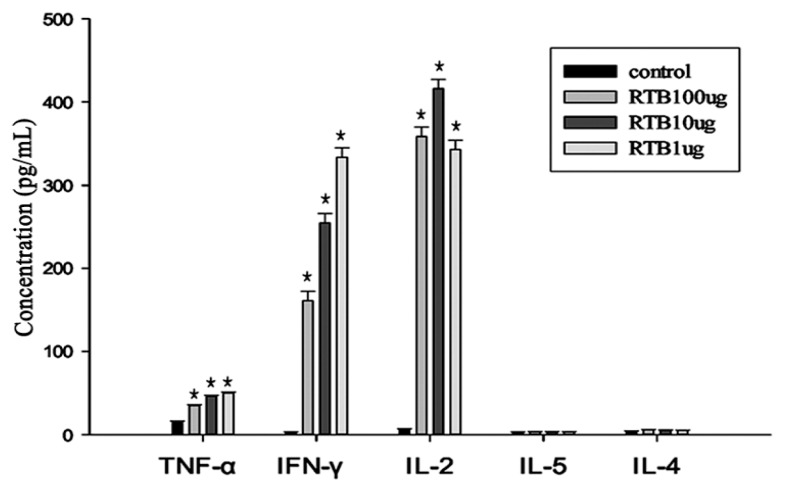
Expression of TNF-α, IFN-γ, IL-2, IL-4 and IL-5 in spleen cells from mice with different concentration of RTB through cytometric bead array immunoassay (CBA) analysis. Splenocytes were stimulated with RTB (1, 10 and 100 μg/mL) in the presence of 2.5 μg/mL ConA for 48 h. The culture supernatants were collected and assessed for cytokine production (TNF-α, IFN-γ, IL-2, IL-5, IL-4) by CBA analysis. Data are presented as the means ± SD of three replicates. *p < 0.01 compared to the control group.

The secretion of the IL-2, IL-4, IFN-γ, IL-5 and TNF-α cytokines was measured using a cytometric bead array immunoassay (CBA). After culturing spleen cells *in vitro*, the highest concentrations of TNF-α, IL-2 and IFN-γ that were induced were significant compared to the concentrations induced by the control group (*p* < 0.01) (Figure 6). TNF-α, IL-2 and IFN-γ are indicators of the Th1 type immune response. No significant differences were observed in the IL-4 and IL-5 cytokine levels produced by experimental and control groups.

## 3. Discussion

RTB is one of the ricin protein subunits. RTB is a lectin protein that binds to a diverse group of membrane-bound sugar moieties, this binding is essential for the RTA subunit of the ricin protein to enter the cell. RTB also mediates the retrograde transport of RTA into the endoplasmic reticulum (ER) [11–13]. RTB can be utilized in the development of an RTB-based vaccine; it can also be used as an RTB-based carrier for heterologous vaccine antigens [14]. Studies have shown that RTB can be successfully used as a carrier fused to other molecules. For example, RTB has been fused to various rotavirus antigens, specifically VP7 [8], P24 [15] and NSP4 [9]. Macrophages play an important role in both innate and adaptive immunity. When induced by inflammatory stimulator, macrophages become activated and release lots of bioactive factors, including NO, several cytokines, such as IL-6, TNF-α and other substances to initiate defense responses [16]. In particular, NO cannot only enhance the activity of macrophages, but also plays an important role in the regulation of various immune responses [17]. IL-6 and TNF-α are important cytokine involved in innate immune responses against pathogens. They can enhance various functional responses and contribute to the systemic effects of inflammation [17–19]. In this study, we found that RTB can induce mouse macrophage cell line RAW264.7 cells to produce NO, TNF-α and IL-6. Therefore, it was indicated that RTB can modulate the cell-mediated immunity and that RTB plays a direct activator role for RAW264.7 cells, and the production of NO and cytokines contribute to immunostimulating activities.

Splenocyte proliferation is an indicator of immunomodulation. We observed that the T-cell and B-cell proliferative response to mitogens was enhanced after RTB stimulation at the concentration of 1 to 100 μg/mL; this finding suggests that RTB may enhance cell-mediated immunity and humoral immunity.

T-helper cells are divided into the Th1 and Th2 subsets based on the profile of their cytokine production and their activation to different immune responses [20]. It is important for protection against a particular pathogen that requires the activation of a Th1 type immune response. Th1 cells mediate cellular immunity that is associated with IL-2, IFN-γ and TNF-α production. Th2 cells stimulate humoral immunity that is associated with IL-4, IL-5, IL-6 and IL-10 production [21,22]. We analyzed the expression of these cytokines using highly sensitive techniques, such as the cytometric bead array immunoassay (CBA), in flow cytometry. CBA technology enables the simultaneous measurement of multiple cytokines in a single sample. We observed this in the microbead-based immunoassay with flow cytometry. In this study, spleen cells secreted high levels of TNF-α, IL-2 and IFN-γ. IL-4 and IL-5 were not produced in response to RTB stimulation. Thus, the induction of Th1 cytokine production occurred at concentrations of RTB that had no effect on Th2 cytokine production. These results suggest that RTB can activate T-cells by upregulating Th1 response, and Th1 cells might be the target cells.

## 4. Experimental Section

### 4.1. Protein

The *E. coli* expression vector plasmid PET28a-RTB was made as described in a previous report with a slight modification [23]. PET28a-RTB was transformed into BL21 cells. The plasmid of interest was inoculated into LB medium containing kanamycin (100 mg/L) and incubated overnight at 37 °C. The culture was induced in LB medium using isopropyl-β-d-thiogalactopyranoside (IPTG) to stimulate recombinant protein synthesis. PET-28a and the sample were passed through a 2 mL nickel column (Ni-NTA; General Electric, Uppsala, Sweden). A recombinant protein with a molecular weight of 34 kD was observed mainly in the inclusion bodies. The inclusion bodies were washed extensively and then solubilized with 6 M urea. The denatured RTB proteins with various concentrations were diluted into renaturation buffer: 0.1 M Tris-HCl (pH 8.0), 0.5 M arginine; the ratio of GSH to GSSG was 0.9/0.1 mM. After incubation at 4 °C for 24 h, the refolded RTB protein was dialyzed to PBS (0.01 M) for 12 h.

The purified protein was confirmed by SDS-PAGE analysis. Then, the protein was passed through Detoxi-Gel endotoxin resin to remove the LPS.

### 4.2. Mice and Cell Line

Female BALB/c mice (4 weeks of age) were purchased from the Changchun H&N Animal Breeding Center. The animals were maintained under specific pathogen-free conditions.

RAW264.7 cells were cultured in RPMI-1640 medium (Gibco, Carlsbad, CA, USA) supplemented with 10% fetal calf serum (FCS) (Hycolone, Logan, UT, USA), 100 U/mL of penicillin and 100 U/mL of streptomycin at 37 °C in a humidified atmosphere of 5% CO_2_.

### 4.3. Cell Viability Assay

Cell viability was determined by alamarBlue assay. RAW264.7 cells were seeded into 96-well plates at a concentration of 4 × 10^4^ cells/well 24 h before treatment. After various concentrations of RTB were added, the cells were then incubated with alamarBlue cell viability reagent (Invitrogen, Frederic, MD, USA) for 48 h at 37 °C. The absorbance was measured at a 570 and 610 nm wavelength.

### 4.4. NO Production Assay

Nitric oxide generated by activated macrophages was determined by Griess reagent (Beyotime, Haimen, China). Nitrite production was measured at the absorbance of 540 nm.

### 4.5. Splenocyte Proliferation Assay

The mice were sacrificed, and spleens were removed aseptically. To measure the extent and type of T-cell response, single cell suspensions of the spleen were cultured in 96-well flat-bottomed plates (Corning) at a concentration of 1 × 10^7^ cells/mL in complete medium containing 10% fetal calf serum. Concanavalin A (ConA, 2.5 mg/mL; Sigma, St. Louis, MO, USA) and LPS (2.5mg/mL; Sigma, St. Louis, MO, USA) were used as the mitogen. Cells were incubated in a humidified incubator at 37 °C (5% CO_2_) for 48 h. After 44 h, 10 μL of MTT (5 mg/mL; Sigma, St. Louis, MO, USA) was added to each well. After 4 h, the absorbance was measured at a 570 nm wavelength. The results were expressed as stimulation index (SI). SI was the absorbance value of stimulated cultures divided by the absorbance value of non-stimulated cultures.

### 4.6. Cytokine Assays

Single cell suspensions from the spleens of non-immunized mice were adjusted to a concentration of 1 × 10^7^ cells/mL in RPMI 1640 complete medium (Gibco, Carlsbad, CA, USA) supplemented with 10% fetal calf serum. Splenocyte cultures were stimulated with 2 μg/mL ConA (Sigma, St. Louis, MO, USA) or ConA plus varying concentrations of RTB antigen for immunostimulation. The production of IL-2, IL-4, IFN-γ, IL-5 and TNF-α was measured from 48 h culture supernatants using cytometric bead arrays (CBA; Becton Dickinson), in accordance with the manufacturer’s instructions. The CBA kit combines beads with the ability to measure the expression levels of IL-2, IL-4, IFN-γ, IL-5 and TNF-α. Data were assayed using a FACSCalibur™ flow cytometer and the Becton Dickinson cytometric bead array (CBA) software [24].

To assess the ability of RTB to induce cytokine production, RAW264.7 cells were stimulated with different concentrations of RTB (1, 10 and 100 μg/mL) and LPS (50 ng/mL) for 24 h. LPS was used as a positive control. TNF-α and IL-6 secretion in the supernatants were measured by ELISA, according to the manufactures’ instructions (Biolegend, San Diego, CA, USA).

### 4.7. Statistical Analysis

Data are representative of three independent experiments, each in triplicate determination. Statistical analysis of the data was performed using one-way ANOVA. The Tukey *post hoc* test was used to determine the significance for all pairwise comparisons of interest. *p*-values of less than 0.05 were considered to represent a statistically significant difference.

## 5. Conclusions

The present study demonstrated the mechanism of RTB activating the immune system. RTB may represent an immunomodulating activation that stimulates macrophages to produce NO, TNF-α and IL-6 and promote Th1-oriented adaptive response. RTB might have the potential for treatment of macrophage-mediated inflammatory disease or act as a potential alternative medicine.
